# Stoichiometric ratios support plant adaption to grazing moderated by soil nutrients and root enzymes

**DOI:** 10.7717/peerj.7047

**Published:** 2019-06-10

**Authors:** Wenjing Ma, Jin Li, Saheed Olaide Jimoh, Yujuan Zhang, Fenghui Guo, Yong Ding, Xiliang Li, Xiangyang Hou

**Affiliations:** 1 Institute of Grassland Research, Chinese Academy of Agricultural Sciences, Hohhot, Inner Mongolia, China; 2 College of Grassland Science and Technology, China Agricultural University, Beijing, China; 3 Key Laboratory of Grassland Ecology and Restoration of Ministry of Agriculture, Hohhot, Inner Mongolia, China

**Keywords:** Nutrient, Stoichiometry, *Leymus chinensis*, *Artemisia frigida*, Enzyme

## Abstract

**Background:**

Vegetation succession is one of the major driving processes of grassland degradation. Stoichiometry significantly contributes to vegetation dynamics. However, a knowledge gap exists in how soil nutrients and root enzymes influence the stoichiometric ratio to affect vegetation dynamics.

**Methods:**

To address these questions, we selected a dominant species (*Leymus chinensis* (Trin.) Tzvel.) and a degraded-dominant species (*Artemisia frigida* Willd.) under different management regimes (enclosure and grazing) on the Inner Mongolia steppe. We measured (i) plant nutrient concentrations, (ii) root enzymes and (iii) soil nutrients to investigate how the selected plant species responded to grazing.

**Results:**

The results show that: (i) N and P concentrations and the C:N:P ratio in different organs are significantly affected by grazing, and there is variation in the plant species’ response. Grazing significantly increased N and P in the leaves and stems of *L. chinensis* and the stems and roots of *A. frigida*. (ii) Grazing significantly increased the activities of glutamine synthase but decreased the activities of acid phosphatase in *L. chinensis*. The nitrate reductase and acid phosphatase activities significantly increased in *A. frigida* under grazing conditions. (iii) Grazing decreased the total nitrogen, total phosphorus, and available nitrogen, but increased the available phosphorus in the soil.

**Conclusion:**

We conclude that *A. frigida* is better adapted to grazing than *L. chinensis,* possibly because of its relatively increased stem and root growth, which enhance population expansion following grazing. Conversely, *L. chinensis* showed increased leaf and stem growth, but suffered nutrient and biomass loss as a result of excessive foraging by livestock, which severely affected its ability to colonize. Root enzymes coupled with soil nutrients can regulate plant nutrients and stoichiometric ratios as an adaptive response to grazing. Thus, we demonstrated that stoichiometric ratios allow species to better withstand grazing disturbances. This study provides a new understanding of the mechanisms involved in grazing-resistance within a plant-soil system.

## Introduction

Grasslands are the largest terrestrial ecosystem in the world and play an extremely important role in the production of food and ecological services for humans. However, continuous overgrazing of grasslands leads to degradation. This not only diminishes productivity, diversity and soil quality but also affects economic growth and ecological sustainability (Pulido et al., 2016; Wang et al., 2017). The Inner Mongolia typical steppe, a major component of the eastern Eurasian temperate steppe and an important production base for animal husbandry in China, faces similar challenges (Li et al., 2012); this is true particularly in areas where the steppe is dominated by *L. chinensis*, which has high palatability, high nutritive value, and high primary productivity (Li et al., 2008). *Leymus chinensis* steppes have degraded into *A. frigida* communities because of long-term grazing during the past several decades (Li, Li & Ren, 2005) and consequently have become characterized by dwarf plants with low productivity. Previous studies have demonstrated that plant adaption to animal grazing depends on plant growth and reproductive characteristics (De Jong & Lin, 2017; Li, Li & Ren, 2005), palatability (Vesk & Westoby, 2001), nutrient use strategies (Hamilton & Frank, 2001), tolerance (regrowth potential after herbivory) (Strauss & Agrawal, 1999) and defence strategies (physical and chemical defence substances) (Zhang et al., 2014). These adaptive characteristics may be attributed to differences in N and P concentrations and the stoichiometric ratios in the plant tissues of different species because of the association of the nutrients with plant growth and ecosystem functions (Elser et al., 2010; Yu et al., 2010).

Ecological stoichiometry is the study of the balance between multiple elements in ecological interactions (Elser et al., 2000a; Elser et al., 2000b). Using this approach, patterns of plant responses to their chemical environment can be well understood. Plant stoichiometry shows why subordinate species withstand drought perturbations (Mariotte, Canarini & Dijkstra, 2017), the trade-off between competitive ability and grazing susceptibility (Branco et al., 2010), and the response of plant species to global-change-driven alterations in resource availability (Yu et al., 2015). The correlation between C:N:P, plant growth, and ecosystem functions (Elser et al., 2010; Yu et al., 2010) is supported by the hypothesis that increasing allocation to P-rich ribosomal RNA supports faster growth rates (Matzek & Vitousek, 2009), and its corollary is related to nutrient use strategies (De Deyn, Cornelissen & Bardgett, 2008) and chemical defence (Royer et al., 2013). The stoichiometric ratio affects the competitive abilities of species under grazing, for example, fast-growing species (competitors) were dominant in a fertilized pasture under low grazing pressure and slow-growing species (tolerant) were relatively abundant in unfertilized grazing systems (Hill et al., 2005). This variation driven by grazing in producer stoichiometry, in turn, can regulate grazing. Earlier reports have shown that animal grazing increased plant N and P concentrations and decreased C:N and C:P ratios on the whole (Bai et al., 2012; Heyburn et al., 2017). However, the rate of nutrient uptake enhanced plant competitiveness but also increased their nutritional quality for herbivores (Branco et al., 2010). Therefore, the stoichiometric ratio responses of plant species to livestock grazing remain latent. Most stoichiometric studies usually focus on the leaf because of its pivotal role in controlling N and P in the carbon obtained (He et al., 2006). However, relatively limited research has been conducted on stems and roots, even though they can serve as nutrient reservoirs that store excess nutrients absorbed from the soil and support the use of N and P in leaves (Cernusak, Winter & Turner, 2010; Yan et al., 2016). In contrast to leaves, the sensitivity of stems and roots to various environments have been demonstrated by woody species in greenhouse studies (Schreeg et al., 2014) and marsh plants along coastlines (Minden & Kleyer, 2014). More importantly, plants respond to grazing by varying the N and P concentrations in leaves, stems and roots as a consequence of changes in the structure and functions of the whole plant (Matzek & Vitousek, 2009). Hence, N and P concentrations and the stoichiometric ratio of stems and roots in response to grazing require further study.

A plant’s C:N:P stoichiometry can be strongly influenced by the environment, despite an organism’s considerable capacity to maintain their body stoichiometry within ranges (Sardans, Rivas-Ubach & Peñuelas, 2012). Soil nutrients can affect the stoichiometric ratios of plants; for example, the C, N and P contents of leaves and litter have been shown to be positively related to soil C, N and P contents (Ordoñez et al., 2009; Yang, Liu & An, 2018). Many studies have found that grazing can alter soil nutrient availability, which is strongly related to dung and urine deposition from grazing animals (Guo et al., 2017), litter decomposition rates (Semmartin et al., 2004), mineralization and nutrient cycling, etc. Moreover, grazing promotes greater accumulation of soil organic carbon (SOC) depending on the root system biomass, which in turn promotes more root biomass, fine root exudates and microbial biomass (Wilson et al., 2018). Previous studies have shown how animal grazing can increase N availability in soils, with an attendant significant decrease in plant above- and below-ground C:N ratios and a subsequent increase in plants below ground N:P ratios (Bai et al., 2012; Zheng et al., 2012). However, unpredictable changes in C:N:P ratios have also been reported, indicating that plant stoichiometry may not be simply related to soil nutrient availability (Heyburn et al., 2017). Therefore, key knowledge gaps exist regarding how long-term grazing influences plant stoichiometry, and whether or not changes in C:N:P ratios in plant tissues might be related to predictable changes in soil C, N, and P contents and storage; this study sought to fill these gaps. Notably, nutrient uptake rates may be regulated by inducible enzymes synthesized by plants. Niklas & Edward (2005) found that plants can adjust N concentration by releasing a certain amount of ribulose bisphosphate carboxylase/oxygenase (Rubisco) in photosynthetic organs. Nitrate reductase and glutamine synthase are two important enzymes in plant nitrogen metabolism (Zioni, Vaadia & Lips, 1971) and serve as the rate-limiting enzymes in the biochemical pathway for nitrogen assimilation. Phosphatase enzymes are responsible for the release of P from organic P-esters, which is one of the P-acquisition strategies (Rejmánková & Macek, 2008). However, little is known regarding the regulation of root enzymes as a grazing response in different plants.

In this study, we hypothesized that plant species have different abilities to take up nutrients under unbalanced N and P availability, and that might support plant adaptation to grazing. Thus, we studied the effects of grazing on C, N, and P contents and stoichiometry in the leaves, stems, and roots of two plant species (*L. chinensis* and *A. frigida*). *Leymus chinensis* is a xeric rhizomatous perennial grass, 0.4–0.9 m in height, that dominates the steppe zone of the Inner Mongolian Plateau, and it is also found in northeast China. *Artemisia frigida* is a semi-shrub that grows on sandy and gravelly soil of the Inner Mongolia Autonomous Region, in the northern part of China and is the dominant species of degraded grassland communities. Specifically, we address three questions: (1): How do the C, N and P concentrations and their stoichiometric ratios in the leaves, stems, and roots of two plant species respond to grazing? (2): How do plants mediate root enzymatic activities in response to grazing? (3): How does grazing induce soil nutrients to moderate species adaption?

## Materials and Methods

### Study sites

The study was conducted at the Inner Mongolia Grassland Ecosystem Research Station (43°38′N, 116°42′E), located in the Xilin River Basin, Xilinhaote, Inner Mongolia Autonomous Region, China. The soil is dark chestnut with a 20–30 cm thick humus layer and a calcic layer below 50–60 cm depth. The region is within a temperate-semiarid climate, with an annual mean temperature of approximately 0 °C and annual precipitation of approximately 350 mm. The precipitation fluctuates from 180 to 550 mm, 60–80% of which falls during the summer season of June to August. The growing season lasts from early April to late September for perennial plant species, whereas annual plants usually germinate in early July following the rains (Li et al., 2016).

### Experimental design and measurements

**Figure 1:**
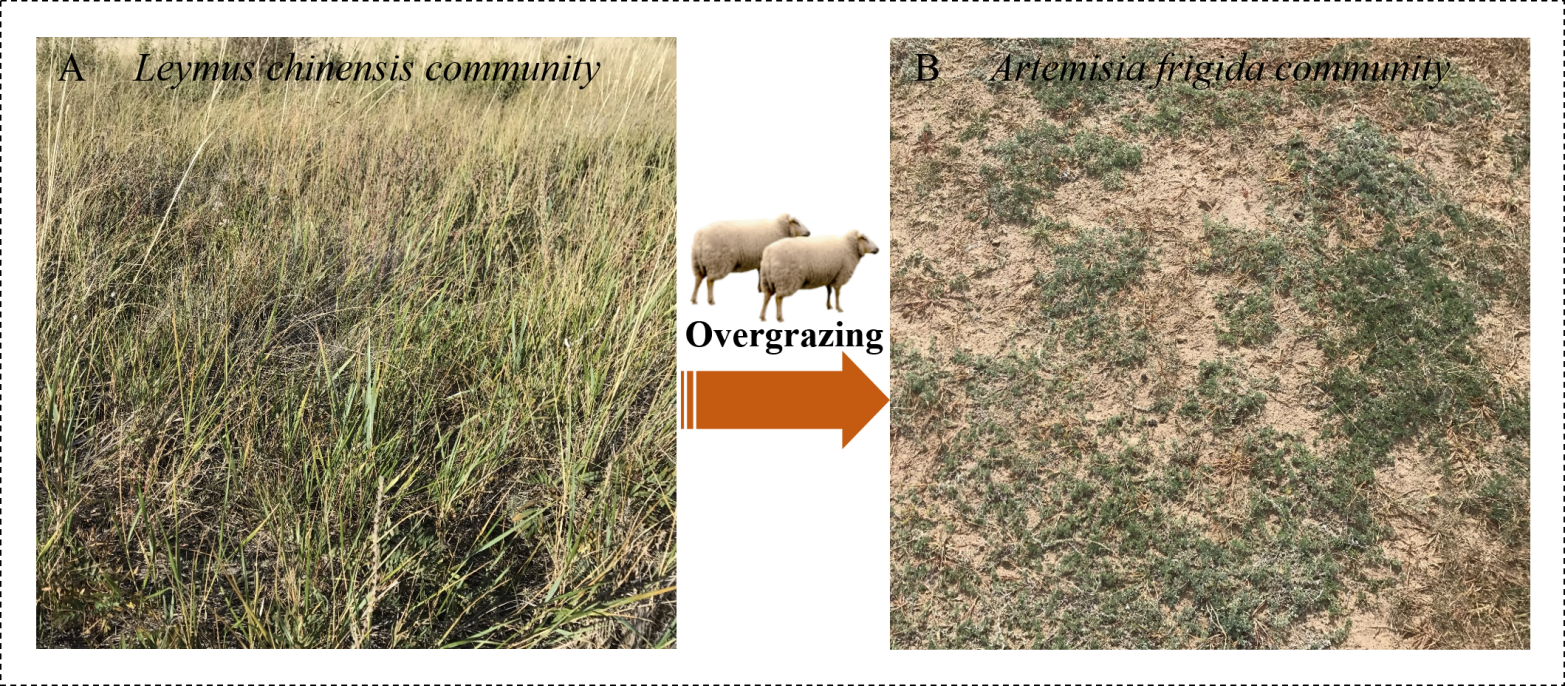
Two communities in Inner Mongolia steppe. A typical community of *Leymus chinensis* located in Xilinhaote, Inner Mongolia Autonomous Region, China. The forms degenerate to B, *Artemisia frigida* community due to long-term grazing as used in this study. Photo credit: Wenjing Ma.

The experiment was conducted at the peak of plant biomass accumulation during the middle of August. Two adjacent plots were selected to reduce the impact of climate factors. The first was an enclosure plot that has been excluded from sheep grazing for 34 years (from 1983 until the year of sampling), which was used as the control plot (C). The second was a grazing plot (G) that has been subjected to continuous free grazing with a stocking rate of approximately 9 sheep equivalent (SE) ha^−1^ year^−1^ with the same duration as the enclosure plot. Grazing begins in early June and ends during mid-October. The two plots have never been fertilized or mowed during the management (Fig. 1). To decrease the spatial variation and soil heterogeneity, we selected the sampling areas 50 × 400 m in size for the control sites and 20 × 400 m in size for the grazing sites. Subsequently, we randomly chose five subplots in each sampling area (0.2 ha and 0.1 ha for the control and grazing plots, respectively) set at intervals of at least 10 m. This study adopts pseudo-replication and a space-for-time substitution limitation (Hurlbert, 1984).

Following the principles of replication and randomization, there were five replicates for *L. chinensis* and *A. frigida* within each subplot; and these were combined and mixed to create one composite sample for each species. Taking *L. chinensis* as an example, we first selected and marked this species, then carefully uprooted the soil core of the whole plant using a shovel (20 cm depth) after removing impurities such as litter and stones. Roots were cautiously removed from the matrix to minimize root loss, repeatedly washed until they were fully exposed and subsequently rinsed with distilled water. Prior to drying, some fresh root samples near the tips (<0.5 mm in diameter for *L. chinensis* and <1 mm in diameter for *A. frigida*) were excised and placed into a 10 ml centrifuge tube and immediately frozen in liquid nitrogen before being taken to the laboratory for storage in a −80 °C cryogenic refrigerator for enzymatic analysis. Soil samples were taken at 0–20 cm depth in each of the soil cores following plant sampling. The plant samples were taken to the laboratory for analysis.

In the laboratory, the plant samples (*L. chinensis* and *A. frigida*) were separated into leaves, stems (containing leaf sheath) and roots (no seeds at the time of sampling), and oven-dried at 65 °C for 48 h to determine the dry matter content. The samples were milled (XL-02A, Xulang, China) and subsequently divided into two parts for determination of C, N and P concentrations in the leaves, stems, and roots. The first part was used to analyse the C concentration using the K_2_Cr_2_O_7_–H_2_SO_4_ oxidation method (Yu et al., 2012a). The second part was digested with acid and used to determine the N and P concentrations. Total nitrogen (N) was measured using the Kjeldahl method, and total phosphorus (P) was analysed using the molybdenum blue colourimetric method (Lü et al., 2015).

Soil samples were air-dried at room temperature for two weeks after removing fine roots and stones. The soil samples were ground, homogenized, and passed through a 2 mm mesh sieve. The total N and P contents of the soil samples were determined as described for the plant samples (Yang et al., 2017). The available nitrogen in the soil was measured using a micro-diffusion technique after alkaline hydrolysis. The soil available phosphorus was analysed using molybdenum antimony blue colourimetry after extraction with NaHCO_3_ (Fu et al., 2000).

The frozen root samples were analysed after 50 days of sampling. The frozen root samples were ground in a freezer grinder (JXFSTPRP-CL, Jingxin, China) to form slurries, and the activities of nitrate reductase (NR), glutamine synthase (GS) and acid phosphatase (ACP) were measured. The activities of NR and GS were assayed according to a test described by Sakar et al. (2010) and (Yu et al., 2012b) respectively. The activity of ACP was determined according to the method described by Wei et al. (2017).

### Statistical analysis

Data were analysed using SPSS 19.0 (SPSS, Chicago, IL, USA). Before applying parametric tests, we tested for the normality and homogeneity of the variances for all data; variables were transformed (ln, sin, cos), if necessary, to meet the assumptions of the analysis of variance (ANOVA). One-way ANOVA was performed to compare the plant nutrient stoichiometry, soil nutrients and root enzymatic activities of the two plant species (*L. chinensis* vs *A. frigida*). Duncan’s multiple range test was used to determine the difference in means at a 0.05 probability level. The effects of treatment and species on the C, N and P concentrations and stoichiometric ratios of the leaves, stems and roots of the plant species were tested using two-way ANOVA. All resulting figures were prepared using Origin 8.5.

## Results

### Differences in nutrient concentrations and stoichiometry of leaves, stems, and roots in the two plant species

We examined the responses of the C, N and P concentrations in the leaves, stems, and roots of two plant species at the control and grazing sites (Tables 1 and 2). The C concentrations in the leaves, stems, and roots were unchanged in both species except that grazing increased the C concentrations in the leaves of *A. frigida*. Treatment × species did not significantly affect the C concentration in each organ. Comparatively, the highest N concentration was observed in the leaves of *L. chinensis* in the grazing plot. N concentration in each organ was not affected by treatment × species except for the leaf. Grazing significantly increased the N concentration in the leaves and stems, but not in the roots of *L. chinensis*. For *A. frigida*, the N in the leaves did not change, but the N concentration in the stems and roots increased at the grazing site, recording a 42.04% and 27.94% increase respectively. There was no significant difference in the P concentration in the leaves between the control and grazing plots for the two species. Treatment × species affected the P concentration in the stems and roots. The P concentration in the stem fractions recorded a higher P concentration at the grazing sites for both species. However, the P concentration was significantly higher for the roots of *A. frigida* under the grazing treatment, but no difference was observed for the same element in the roots of *L. chinensis*.

**Table 1:** Effects of treatment, species, and their interaction on the C, N and P concentrations and stoichiometric ratios of leaves, stems and roots of two plant species.

	Treatment		Species		Treatment * Species	
	*F*	*Sig*	*F*	*Sig*	*F*	*Sig*
Leaf C	0.74	0.40	0.48	0.25	1.18	0.29
Stem C	1.95	0.18	0.70	0.41	2.27	0.15
Root C	0.20	0.66	10.69^*^	0.00	0.04	0.84
Leaf N	30.94^*^	0.00	0.00	0.98	22.68^*^	0.00
Stem N	18.77^*^	0.00	1.59	0.23	0.14	0.71
Root N	7.90^*^	0.01	3.53	0.08	1.10	0.31
Leaf P	2.75	0.12	84.38^*^	0.00	2.97	0.10
Stem P	130.54^*^	0.00	0.08	0.79	8.15^*^	0.01
Root P	14.92^*^	0.00	9.31^*^	0.01	13.24^*^	0.00
Leaf C:N	18.81^*^	0.00	0.26	0.62	31.40^*^	0.00
Stem C:N	11.73^*^	0.00	0.01	0.92	3.73 × 10^−4^	0.98
Root C:N	1.53	0.23	9.55^*^	0.01	0.52	0.48
Leaf C:P	1.90	0.19	35.39^*^	0.00	5.16^*^	0.04
Stem C:P	96.15^*^	0.00	0.05	0.83	6.54^*^	0.02
Root C:P	3.80	0.07	11.65^*^	0.00	6.72^*^	0.02
Leaf N:P	7.31^*^	0.02	82.24^*^	0.00	5.16^*^	0.04
Stem N:P	18.62^*^	0.00	0.01	0.91	7.16^*^	0.02
Root N:P	0.52	0.48	0.46	0.51	4.98^*^	0.04

**Notes.**

Treatments are control plot and grazing plot. *F*-values and significance levels of two-way ANOVA are shown.

* *P* < 0.05.

**Table 2:** C, N and P concentrations of leaves, stems and roots of two plant species in control plot and grazing plot.

		*Leymus chinensis*		*Artemisia frigida*	
		Control	Grazing	Control	Grazing
C (g/kg)	Leaf	292.18 ± 22.09^a^	295.83 ± 25.78^a^	302.24 ± 10.06^b^	331.42 ± 6.15^a^
	Stem	221.12 ± 12.96^a^	218.98 ± 16.54^a^	198.90 ± 5.45^a^	199.60 ± 3.99^a^
	Root	203.99 ± 3.75^a^	222.09 ± 14.53^a^	181.51 ± 6.52^a^	190.92 ± 3.61^a^
N (g/kg)	Leaf	18.50 ± 0.75^b^	29.99 ± 1.54^a^	23.83 ± 1.06^a^	24.72 ± 0.95^a^
	Stem	10.30 ± 0.94^b^	15.02 ± 1.37^a^	9.42 ± 0.95^b^	13.38 ± 0.58^a^
	Root	8.88 ± 0.51^a^	10.09 ± 0.58^a^	9.45 ± 0.57^b^	12.09 ± 0.97^a^
P (g/kg)	Leaf	0.48 ± 0.03^a^	0.62 ± 0.06^a^	0.94 ± 0.04^a^	0.94 ± 0.09^a^
	Stem	0.45 ± 0.03^b^	0.74 ± 0.03^a^	0.35 ± 0.03^b^	0.82 ± 0.04^a^
	Root	0.46 ± 0.03^a^	0.46 ± 0.03^a^	0.44 ± 0.03^b^	0.69 ± 0.04^a^

**Notes.**

a,b Rows with different superscript are significantly different ( *P* < 0.05) for each plant species. Mean ± standard errors.

**Figure 2:**
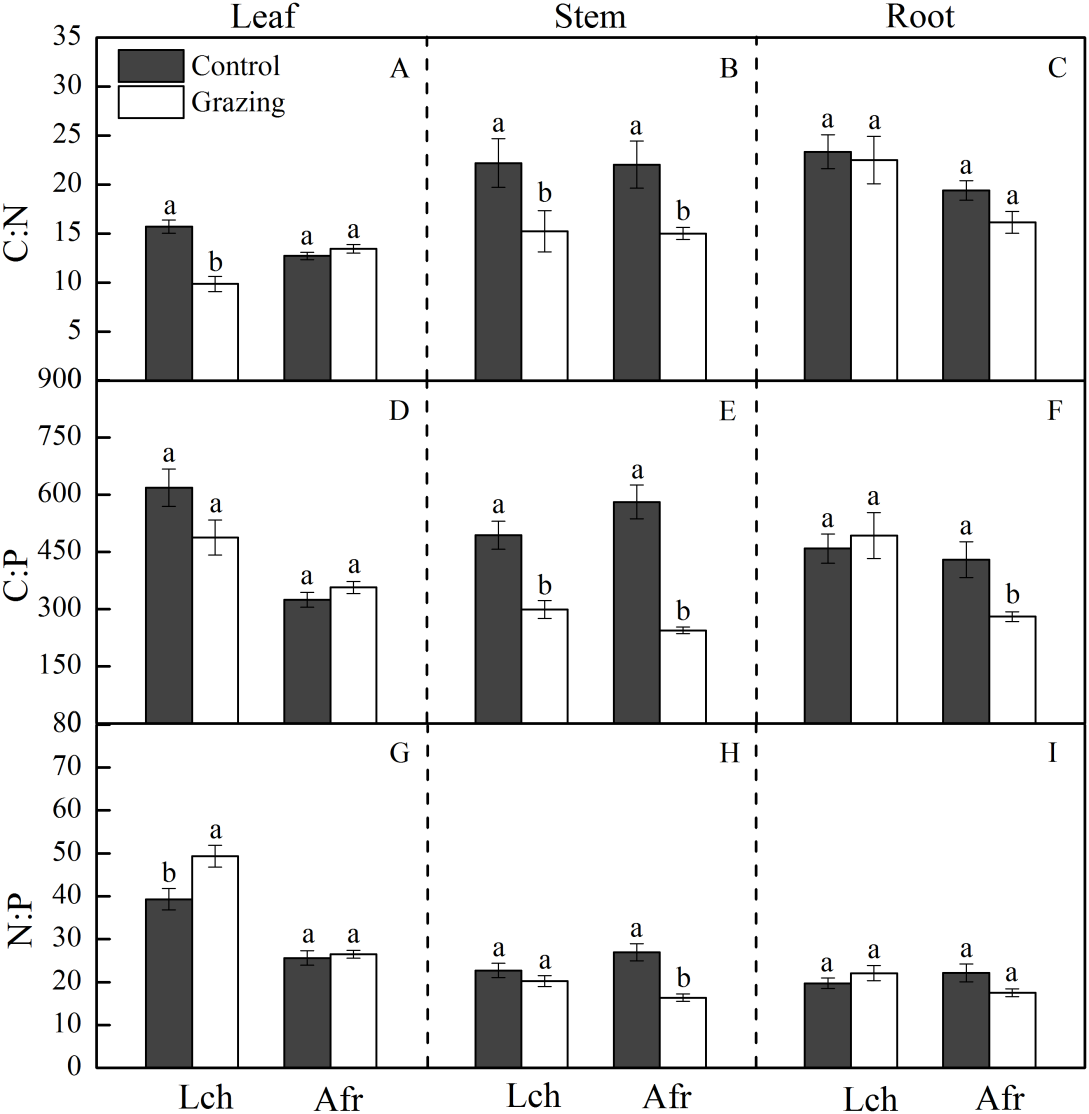
Effect of grazing on C, N and P stoichiometric ratios in the two plants. The bars are mean ± standard errors. Error bars show the standard errors of five replicates. Lch is *Leymus chinensis*, Afr is *Artemisia frigida*. (A) is C:N ratios in the leaf, (B) is C:N ratios in the stem, (C) is C:N ratios in the root, (D) is C:P ratios in the leaf, (E) is C:P ratios in the stem, (F) is C:P ratios in the root, (G) is N:P ratios in the leaf, (H) is N:P ratios in the stem and (I) is N:P ratios in the root. Different letters indicate significant differences (*P* < 0.05) based on one-way ANOVA.

The C, N and P stoichiometric ratio in the leaves, stems, and roots of *L. chinensis* and *A. frigida* in response to grazing are shown in Table 1 and Fig. 2. Grazing significantly decreased the C:N ratio in the leaf and stem of *L. chinensis*, but no change was observed in the C:N ratio for the root. Similarly, the C:N ratio in the stem fraction of *A. frigida* decreased as a result of grazing, while that in the leaf and root of the plant remained unchanged. Treatment × species did not significantly affect the C:N ratio in each organ except the leaf. For *L. chinensis*, the stem C:P ratio significantly decreased as a result of grazing, while the C:P ratio in the leaf and root remain unchanged. For *A. frigida*, grazing reduced the C:P ratio in the stem and root but not the leaf. The C:P ratio in the leaf and root of *L. chinensis* were significantly higher than that of *A. frigida*, but that was not true for the stem. Treatment × species significantly affected the C:P ratio in each organ. The N:P ratio in the leaf of *L. chinensis* was significantly increased by grazing, and N:P ratio in the stem and root remained unchanged. Grazing significantly diminished N:P ratio in the stem and had no effect in the leaf and root of *A. frigida.* There was no significant difference in the N:P ratio of the stem and root for both species, while the N:P ratio in the leaf of *L. chinensis* was significantly higher than that of *A. frigida*. Treatment × species significantly affected the N:P ratio in each organ.

### Grazing affects activities of root enzymes

**Figure 3:**
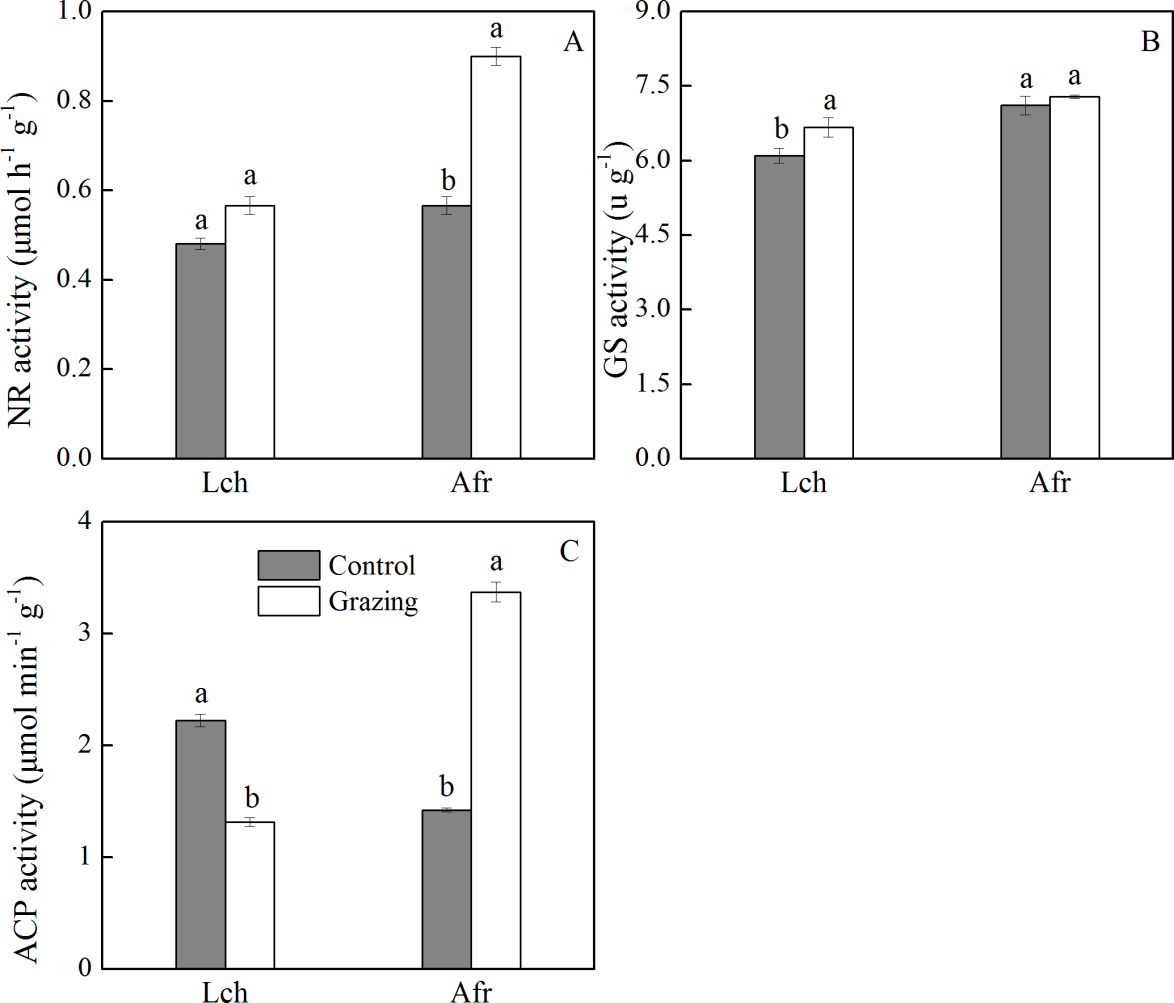
Effect of grazing on root enzymes of two species. (A) Nitrate reductase activity; (B) glutamine synthase activity; (C) acid phosphatase activity. Lch is *Leymus chinensis*, Afr is *Artemisia frigida*. The results of one-way ANOVA for a comparison between the control and grazing for the species. Letters above the columns indicate significant differences (*P* < 0.05).

The effect of grazing on the activities of nitrate reductase, glutamine synthase, and acid phosphatase in the root of *L. chinensis* and *A. frigida* is shown in Fig. 3. The nitrate reductase activity of *L. chinensis* in the two plots showed no significant difference. The nitrate reductase activity of *A. frigida* in the grazing plot was significantly higher than it was in the control plot, as well as what was observed in the control and grazing plots of *L. chinensis*. The glutamine synthase activity of *L. chinensis* in the grazing plot was significantly higher compared to that in the control plot, while the glutamine synthase activity of *A. frigida* was not significantly different between the control and grazing plots. Grazing significantly decreased the acid phosphatase activity of *L. chinensis* (1.32 µmol/ (min g)) compared to that of the control plot (2.22 µmol/ (min g)). The acid phosphatase activity of *A. frigida* in the control plot was lower than that recorded in the grazing plot; the values were 1.42 µmol/ (min g) and 3.37 µmol/ (min g) respectively.

### Grazing affects soil nutrients

**Figure 4:**
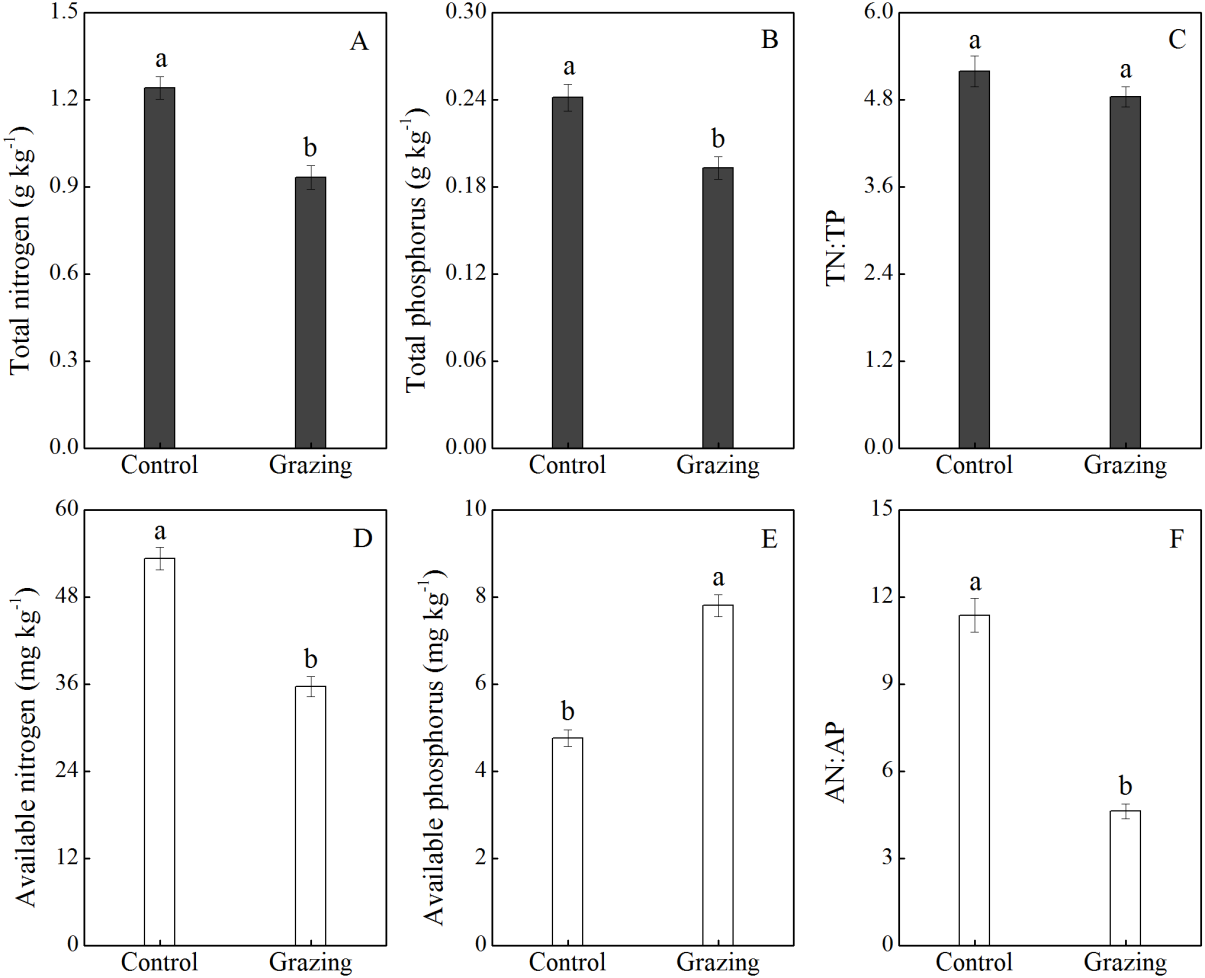
Effect of grazing on N and P nutrient and stoichiometric ratios in the soil. (A) Total nitrogen; (B) total phosphorus; (C) the ratio of total nitrogen to total phosphorus; (D) available nitrogen; (E) available phosphorus; (F) the ratio of available nitrogen to available phosphorus. Different letters represent significant differences among treatments at a 95 % confidence interval.

The effects of grazing on soil nutrients are shown in Fig. 4. Grazing decreased the soil total N and total P, with a mean reduction of 25.00% and 20.83% respectively. There was no significant difference in the ratio of soil total N to total P between the control plot (5.19) and the grazing plot (4.84). In addition, available nitrogen was higher in the control plot (53.30 mg/kg) than in the grazing plot (35.68 mg/kg). Conversely, available phosphorus was significantly higher in the grazing plot. Compared to the control plot, the available phosphorus increased by 63.87% in the grazing plot. The changes in available nitrogen and available phosphorus resulted in a decreased AN:AP ratio in the soil under the grazing treatment.

## Discussion

### Implication of C, N and P concentrations and stoichiometry in different organs

Herbivore grazing may modify plant C, N and P concentrations and stoichiometry. Bai et al. (2012) showed that grazing increased N content in the leaves of plant species in three community types: meadow, typical and desert steppe. In this study, grazing enhanced N concentrations in the leaf of *L. chinensis*, but it did not in *A. frigida*. This finding supports that changes in N and P concentrations because of grazing are species-specific, as reported by Yang et al. (2017) that grazing did not change the N content of *Stipa grandis* but influenced the N content of *Agropyron cristatum*. Elsewhere, Li et al. (2010) also found that N and P concentrations for several species were not affected by grazing, but N and P concentrations in *Poa pratensis* were higher under grazing treatments than in control treatments. Most previous studies have focused on leaf nutrient concentrations and stoichiometry because of its role in obtaining carbon by controlling N and P. Zheng et al. (2012) discussed how stoichiometric changes in root tissues respond to grazing, with a consequent increase in N and P contents in the roots. Our data found that grazing enhanced N and P concentrations in the root tissue of *A. frigida*. Interestingly, we observed an increase in N and P concentrations in the stem of the two plant species, showing a sensitive response by this organ to grazing. These results agree with Minden & Kleyer (2014) and Schreeg et al. (2014) that the stem and belowground organs show a higher variation in elemental composition than the leaf in herbaceous, semi-shrub and marsh species. In addition, our result corroborates the evidence put forward by Zheng et al. (2012) that the leaf is not the only plant organ that responds to grazing, but the stem and root also show a response.

Plant growth requires proteins synthesis, which in turn demands large amounts of N and P for the ribosome; thus, fast-growing species are characterized by low C:P and N:P ratios (Elser et al., 2000b). Therefore, variations in stoichiometric ratios in plant organs have significant implications for the fundamental ecological process of a grazing response (Elser et al., 2010; Peñuelas et al., 2013). In our study, the increase in N and P concentrations in the leaf and stem suggest that both the leaf and stem growth of *L. chinensis* are positively influenced by grazing. This agrees with the result that *L. chinensis* responds to grazing by improving compensatory growth to reduce biomass loss (Wang et al., 2004). Another experiment by Niu, He & Lechowicz (2016) reported that plants in Tibetan alpine meadows increase foliar nutrients but tend to have lower leaf dry matter content, which accelerates growth and regrowth. However, it is noteworthy that compensatory growth is directly linked to grazing intensity. Zhao, Chen & Lin (2008) showed that *L. chinensis* exhibited over-compensatory growth at light and moderate clipping intensities (20% and 40% aerial mass removed) with a greater accumulated aboveground biomass; however, intense clipping (80% aerial mass removed) removed most of the aboveground tissues, which greatly reduced the growth of aboveground biomass and resulted in under-compensatory growth compared to that of the unclipped plants. Therefore, plants may not resist the damage caused by overgrazing if the amount of livestock foraging is more than the increase in biomass. However, *A. frigida* shows a different adaptive strategy that is devoid of change in leaf growth rate, but rather it uses an increase in its stem and root growth rates under grazing. Orians, Thorn & Gómez (2011), using a conceptual model for resource flow in plants, proposed that herbivore-induced export of nutrients from fine roots into stems and storage roots sequestered nutrients in tissues inaccessible to herbivores. This finding concurs with the results of Li, Li & Ren (2005) that grazing enhanced branch and indefinite root density of *A. frigida* as an adaptive strategy to grazing at a moderate stocking rate (4.0 sheep ha^−1^) in a rotational grazing system. In addition, the adaptation of *A. frigida* to grazing was supported by the consequent population expansion resulting from the rapid growth of the stem and root (Hou et al., 2009).

A higher foliar C:N nutrient ratio allows for increased C storage per unit nutrient, i.e., high C:N and C:P ratios represent high utilization of N and P by plants (Elser et al., 2010; Peñuelas et al., 2013). Thus, species with slow growth and a conservative resource-use strategy may dominate nutrient-limited soils by enhancing resource use efficiency (with a minimal investment of ribosomes per unit protein synthesized). In contrast, under high-resource conditions where rapid growth and acquisitive resource-use strategy are a better competitive advantage, higher investment in ribosomes per unit of protein maximizes the speed of protein synthesis and therefore growth (Bai et al., 2012; Sardans & Peñuelas, 2013). For example, Minden & Kleyer (2014) found that species of a frequently inundated marsh (nutrient-rich) had a lower C:N ratio than that of a nutrient limiting marsh (nutrient-poor). The C:N ratio in the leaf of *L. chinensis* decreased but remained unchanged in the leaf of *A. frigida*, and this indicated that the latter possesses a conservative resource-use strategy under grazing. The importance of suitable leaf stoichiometry for dominance at grazing sites is also reflected in the chemical anti-herbivore defence (Endara & Coley, 2011). Royer et al. (2013) found that C-based secondary defensive compounds are significantly and positively correlated to C:N. The authors further noted that C:N ratio can be considered a good indicator of the secondary compound concentration in organs, particularly for those involved in chemical defence. The leaf C:N ratio of *A. frigida* is higher than that of *L. chinensis* indicating that *A. frigida* may be appropriate to prevent defoliation by herbivores because of its higher level of defensive substance. This is supported by Liu et al. (2015) who reported that moderate and severe mechanical damage rapidly increase the secondary metabolites of *A. frigida* and the main components of secondary metabolites are terpenoids, which can inhibit the rate and time of defoliation. Da Silveira Pontes et al. (2015) reported that herbivores preferred to eat tender leaves of fast-growing species with rich nitrogen rather than the leaves of slow-growing species, which are richer in complex carbon compounds not involved in photo assimilation. Therefore, leaf nutrients and stoichiometry maximize plant fitness in a particular environment, reflecting a different trade-off between growth and defensive ability (Sardans & Peñuelas, 2013). In our study, *L. chinensis* increased its growth rate to compensate for biomass loss, with a consequent limit in its defensive ability because of the increase of N in the leaf of *L. chinensis*, which improves its herbivore palatability. However, *A. frigida* may have a better capacity to either tolerate or avoid defoliation at the expense of leaf growth.

### Root enzymes may regulate plant adaption to grazing

A plant stoichiometric ratio may be regulated by synthesized enzymes, with possible changes in the rate of nutrient uptake. The correlation between nitrogen and phosphorus availability with root and rhizosphere enzymes has been proved (Raynaud, Jaillard & Leadley Paul, 2008; Rejmánková & Macek, 2008). Nitrate reductase and glutamine synthase are two important enzymes in plant nitrogen metabolism. The first step of nitrate degradation occurs in the cytoplasm, where nitrate reductase converts nitrate to nitrite. Subsequently, ammonia and glutamine degraded by nitrite reductase form glutamine through glutamine synthase (Campbell & Kinghorn, 1990). Previous research has found that nitrification enzymatic activity is significantly influenced by the management regime (grazing and mowing) (Patra et al., 2006), suggesting that eubacterial structures and free-living N^2^-fixing communities are controlled by management, whereas the diversity of nitrate reducers and ammonia oxidizers is dependent on both management and plant species. In our study, observations showed that *L. chinensis* and *A. frigida* have different pathways of enhancing nutrient utilization to increase the N concentration. *Leymus chinensis* slightly improved the assimilation of ammonium nitrogen in its roots by increasing the GS activity but showed no increase in nitrate assimilation. *Artemisia frigida* increased nitrate nitrogen assimilation by greatly increasing NR activity. Acid phosphatases are important components of the response of plants to P limitation (Ciereszko et al., 2017). Acid phosphatase, an induced enzyme, catalyses organophosphorus (present both in soil and plant tissues) into small molecules of available phosphorus and functions in the processes of P uptake, transport and recycling (Ciereszko et al., 2017; Duff, Sarath & Plaxton, 1994). The increase in acid phosphatase activity under P-deficient conditions has been documented for various plant species, including wheat, lupine and clover, or sedges (Carex) (Ciereszko et al., 2017; Güsewell, 2017; Hunter & McManus, 1999). Our results indicate that grazing decreased the ACP activity in the root of *L. chinensis* suggesting that its growth is not restricted by phosphorus under grazing. The decrease in ACP activity may have been because the grazing increased available P in the soil, as a way of meeting the P required for the growth of *L. chinensis.* However, *A. frigida* increased the ACP activity when the soil available P increased, which indicated that *A. frigida* may require more P for its growth compared to that of *L. chinensis.* This result supports Liu, Tian & Zhang (2014) that *A. frigida* has a high physiological demand for phosphorus. In addition, it has great potential to hydrolyse and release organophosphorus in the soil for plant root growth, thereby increasing its colonization. As indicated by our results, *A. frigida* increased the N and P concentration by increasing the NR and ACP activities to regulate, which may have supported its adaptation to grazing by promoting the growth of the stems and roots.

### Changes in soil nutrients induced by grazing

Grazing-induced soil properties can control the variation in C:N:P stoichiometry in plant tissues, which may be related to climate, steppe type, grazing intensity, and livestock species (Li et al., 2010; Wu et al., 2011). In our study, grazing decreased total N, total P, and available N while it increased available P in the soil. Long-term grazing leads to nitrogen loss in grassland ecosystems because of the cumulative effect of the continuous removal of soil total nitrogen from the ecosystem by foraging animals, although animals only use 10–15% of the N ingested and the remaining is excreted in urine and dung (Duff, Sarath & Plaxton, 1994). In addition, the N-fixation process is limited by the decrease in the number of leguminous plants. The effect of grazing on soil available N is mainly related to many complex factors and processes, such as plant litters, microorganisms, soil properties, litter decomposition, soil respiration, and N mineralization. This process is mainly caused by the selection of high-quality pasture by herbivores, which increases the abundance of inferior plants (lower N content or organic compounds in chemical defence), reduces the litter quality and slows the decomposition rate, thereby reducing the available nitrogen content in the soil (Abbasi & Adams, 2000). Grazing reduces the quantity and quality of litter, leading to a decreased input of soil organic matter pools and C supply from microorganisms (Golluscio et al., 2009), which in turn results in a negative effect on soil C and N cycles. Stable isotope tracers found that grazing decreased ^15^N recovery both in plant and microbial N pools but strongly promoted NO_3_^−^ accumulation in soil thus negatively affecting potential ecosystem N retention (Wu et al., 2011). In addition, grazed areas may be subject to an additional loss of N-rich topsoil through wind erosion (Steffens et al., 2008). For phosphorus, the results from the research described here provide evidence that grazing reduces soil total P content. A study conducted on the effect of grazing on soil total P in a *L. chinensis* steppe after 19 years of grazing found that in the 0–60 cm soil layer, 24.9% of soil P stock was lost, mainly in the organic form (Sternberg et al., 2015). In this study, we observed that soil available phosphorus increased in the grazing plot compared to that of the control plot. This is consistent with Rui et al. (2012), who found that free grazing significantly increased APi (an inorganic form of phosphorus in the soil extracted by the extractant solution of 1 M ammonium chloride (NH_4_Cl)) and BPi (an inorganic form of phosphorus in the soil extracted by 0.5 M sodium bicarbonate (NaHCO_3_)) in the 0–10 and 10–20 cm soil layers respectively. In natural ecosystems, the decomposition of feces and urine excreted by herbivores is a faster pathway to release available P. Microbial mineralization of organic P is a key process of soil P cycling in unfertilized/natural ecosystems to produce available inorganic P for plants (Chen et al., 2004), which may be affected by grazing and a grazing-induced increase in soil temperature because of enhanced solar radiation. Therefore, grazing can stimulate microbial activity and have an impact on plant root exudation and mycorrhizal fungi, which can further stimulate the excretion of phosphatase and organic acids to release P (Oburger et al., 2009).

Previous studies have found that animal grazing increases N availability in soils with an attendant significant decrease in plants above- and below-ground C:N ratios, as well as an increase in plant belowground N:P ratios (Bai et al., 2012; Zheng et al., 2012). In our study, we observed that grazing decreased the soil available N but increased plant N, consistent with a previous report that grazing decreases soil available N (Chen et al., 2018). The decrease of available N in the soil increases the limiting effect of the element on the growth of *L. chinensis*. This concurs with Bai et al. (2014), who found that the growth of *L. chinensis* may be subject to N limitation during dry years. A nitrogen addition experiment conducted in a temperate climate and in a plot fenced to exclude livestock grazing showed a linear decrease in the biomass of *A. frigida* with an increase in N addition. This suggests that N has little effect on the growth of *A. frigida* (Fang et al., 2012). Therefore, the reduction in N available in the soil had less effect on *A. frigida* compared to that on *L. chinensis*. Notably, an increase in the available P in the soil as a result of animal grazing could increase the P in plant tissues. Since *A. frigida* is sensitive to phosphorus (Liu, Tian & Zhang, 2014), the increased available P in the soil had a more positive impact on the growth of *A. frigida*. In contrast, the decrease in available N in the soil inhibited the growth of *L. chinensis*. Therefore, changes in nutrient availability induced by grazing affected the nutrient concentration and stoichiometric ratios in plant tissues and the adaptation of the plants to grazing.

## Conclusions

In conclusion, we contend that *A. frigida* is better adaptated to grazing than *L. chinensis,* possibly due to its increased stem and root growth, which enhances its population expansion and its capacity to avoid defoliation by herbivores given its higher level of defensive substance. However, *L. chinensis* increased its leaf growth but was subject to biomass loss as a result of excessive foraging by livestock, severely affecting its ability to colonize. We also found a sensitivity of the stems and roots of herbaceous vegetation to various environments. Root enzymes coupled with soil nutrients can regulate plant nutrients and stoichiometric ratios as an adaptive response to grazing. This result provides a new understanding of the mechanisms involved in grazing-resistance within a plant-soil system. Further studies are required to demonstrate how soil nutrients in the rhizosphere and enzymes in the roots and rhizosphere of plants respond to grazing.

##  Supplemental Information

10.7717/peerj.7047/supp-1 File S1 C, N and P concentrations and stoichiometric ratios of leaves, stems and roots and root enzymes of two plant species in enclosure and grazing plots NR is nitrate reductase, GS is glutamine synthase and ACP is acid phosphatase

10.7717/peerj.7047/supp-2 File S2 Nitrogen and phosphorus nutrient and stoichiometric ratios in the soil N is nitrogen, P is phosphorus

## Additional Information and Declarations

### Data Availability

The following information was supplied regarding data availability: The raw measurements are available as Files S1 and S2. The raw data show two species’ different responses to grazing and how grazing affects soil nutrients. These data were used for statistical analysis to compare species A and species B.
